# Efficacy and Safety of Closure‐Focused Anti‐Reflux Mucoplasty Compared With Conventional ARMS: A Propensity Score–Matched Study (With Video)

**DOI:** 10.1111/den.70201

**Published:** 2026-06-15

**Authors:** Kazuki Yamamoto, Nikko Theodore V. Raymundo, Kohei Shigeta, Yuta Tamaru, Kei Ushikubo, Yohei Nishikawa, Ippei Tanaka, Mayo Tanabe, Kazuya Sumi, Haruhiro Inoue

**Affiliations:** ^1^ Digestive Diseases Center Showa Medical University Koto Toyosu Hospital Tokyo Japan; ^2^ Institute of Digestive and Liver Diseases St. Luke's Medical Center Taguig Philippines

**Keywords:** endoscopic mucosal resection, endoscopy, gastroesophageal reflux disease, non‐erosive reflux disease, proton pump inhibitors

## Abstract

**Background:**

Endoscopic anti‐reflux therapies, including anti‐reflux mucosectomy (ARMS) and mucosal ablation (ARMA), offer minimally invasive treatment for gastroesophageal reflux disease (GERD) but carry risks of stenosis and delayed bleeding. Closure‐focused techniques—anti‐reflux mucoplasty (ARM‐P) and ARM‐P with valve (ARM‐P/V)—were developed to reduce these events, yet no comparative evaluation exists. Therefore, we aimed to clarify whether closure‐focused techniques reduce adverse events while preserving efficacy.

**Methods:**

Patients undergoing first‐time endoscopic anti‐reflux therapy at a single‐center university hospital between 2012 and 2025 were retrospectively analyzed. Procedures were categorized as conventional (ARMA, ARMS) or closure‐focused (ARM‐P, ARM‐P/V). A 1:1 propensity score–matched cohort was created using preoperative clinical and endoscopic variables. Primary outcomes were adverse events; secondary outcomes were symptom scores, Hill grade, and discontinuation of acid‐suppressive therapy. Treatment effects were estimated using average treatment effect on the treated (ATET).

**Results:**

Among 251 patients, 94 matched pairs were generated. Adverse events occurred in 5.32% of closure‐focused cases versus 25.53% with conventional techniques (*p* < 0.001). ATET showed a 40.4% reduction in overall adverse events (*p* = 0.009) and a 30.8% reduction in stenosis (*p* = 0.018). At 2 months, acid‐suppressive medication was discontinued in 45.74% of closure‐focused patients and 35.11% of conventional patients (*p* = 0.266), with comparable symptomatic and anatomical outcomes, but a longer procedure time.

**Conclusion:**

Closure‐focused techniques improve safety without compromising clinical or anatomical outcomes and represent a promising refinement of endoscopic anti‐reflux therapy; however, these findings should be validated in prospective comparative studies.

**Abbreviations**
AET: acid exposure time
AGREE: adverse events in GI endoscopy
APC: argon plasma coagulation
ARMA: anti‐reflux mucosal ablation
ARM‐P: anti‐reflux mucoplasty
ARM‐P/V: anti‐reflux mucoplasty with valve
ARMS: anti‐reflux mucosectomy
ASA‐PS: American Society of Anesthesiologists Physical Status
ATET: average treatment effect on the treated
BMI: body mass index
EMR‐C: endoscopic mucosal resection with a cap
ESD: endoscopic submucosal dissection
FSSG: Frequency Scale for the Symptoms of GERD
GEFV: gastroesophageal flap valve
GEJ: gastroesophageal junction
GERD: gastroesophageal reflux disease
GERD‐HRQL: GERD‐Health Related Quality of Life Questionnaire
GerdQ: Gastroesophageal Reflux Disease Questionnaire
HRM: high‐resolution manometry
LA: Los Angeles
MII‐pH: multichannel intraluminal impedance‐pH
MNBI: mean nocturnal baseline impedance
NERD: Non‐Erosive Reflux Disease
P‐CAB: potassium‐competitive acid blocker
POD: postoperative day
PPI: proton pump inhibitor
SCJ: squamocolumnar junction
SD: standard deviation

## 1 Introduction

Gastroesophageal reflux disease (GERD) is a common and increasingly prevalent disorder of the upper gastrointestinal tract [1]. First‐line management typically includes acid‐suppressive therapy, prokinetic agents, and lifestyle modification [2, 3, 4]. When these fail, surgical fundoplication has traditionally been considered the most reliable treatment option [5, 6, 7].

More recently, endoscopic anti‐reflux therapies have emerged as minimally invasive alternatives that enhance the anti‐reflux barrier through mucosal remodeling at the gastroesophageal junction (GEJ) [8, 9, 10, 11, 12, 13]. Among these, anti‐reflux mucosectomy (ARMS) [14, 15] and mucosal ablation (ARMA) [16, 17] are widely adopted. Both create controlled mucosal injury involving three‐quarters to four‐fifths of the gastric cardia, which heals with contraction to reconstruct the gastroesophageal flap valve (GEFV). These techniques require no specialized equipment and have demonstrated durable efficacy. Meta‐analyses report clinical response rates of 70%–86% and proton pump inhibitor (PPI) discontinuation in up to 55% [18, 19, 20]. ARMS is reimbursed in Japan with benefits reported for up to 5 years, [21] and ARMA has demonstrated durability for at least 3 years [22]. However, recent sham‐controlled studies have reported no significant benefit of ARMA compared with sham procedures, highlighting ongoing controversy in this field [23].

Nevertheless, ARMS and ARMA rely on natural ulcer healing and may lead to delayed adverse events, particularly transient stenosis and delayed bleeding, reported in 7%–14.4% and up to 8.8% of cases [14, 16, 18, 19, 20]. To address these limitations, closure‐focused approaches—anti‐reflux mucoplasty (ARM‐P) [24, 25] and its modified form with mucosal valvuloplasty (ARM‐P/V)—have recently been developed [26, 27]—were developed. These techniques involve a smaller mucosal resection followed by immediate closure of the defect. Pilot studies suggest improved safety while maintaining efficacy comparable to conventional approaches [24, 27]. However, no prior study has directly compared closure‐focused and conventional techniques using matched cohorts.

This study was therefore designed to provide the first comparative evaluation of conventional endoscopic anti‐reflux techniques and the closure‐focused procedure. The aim of this study was to determine whether closure‐focused techniques reduce adverse events and preserve therapeutic effectiveness compared with established conventional procedures.

## 2 Materials and Methods

### 2.1 Study Design

This retrospective cohort study included patients undergoing first‐time endoscopic anti‐reflux therapy at a single university hospital between April 2012 and April 2025. The study was approved by the Institutional Review Board (2025‐0397). Treatment strategies evolved over time, with ARMS introduced in 2012, ARMA in 2018, and closure‐focused techniques introduced in 2022 with ARM‐P, followed by the modified technique ARM‐P/V in 2024. Accordingly, procedure selection largely reflected the chronological development of these techniques. All procedures were performed under general anesthesia by board‐certified expert endoscopists with extensive experience in therapeutic endoscopy. For analysis, ARMA/ARMS were classified as conventional techniques and ARM‐P/ARM‐P/V as closure‐focused techniques. To minimize baseline differences, a 1:1 propensity score–matched analysis was performed.

### 2.2 Primary and Secondary Outcome

The primary outcome was safety, assessed by transient stenosis and delayed bleeding. Transient stenosis was defined as dysphagia requiring endoscopic balloon dilation [16]. Balloon dilation was performed based on clinically significant dysphagia that interfered with oral intake, as assessed by expert endoscopists. Delayed bleeding was defined as hemorrhage requiring endoscopic hemostasis, transfusion, or a decrease in hemoglobin of ≥ 2 g/dL [28]. Other adverse events were graded according to the adverse events in GI endoscopy (AGREE) classification for endoscopic procedures [29]. Secondary outcomes included symptom improvement assessed by the GERD‐Health Related Quality of Life Questionnaire (GERD‐HRQL), [30] the Gastroesophageal Reflux Disease Questionnaire (GerdQ), [31] and the Frequency Scale for the Symptoms of GERD (FSSG) [32] score, endoscopic changes in GEFV morphology using the Hill classification, and changes in acid‐suppressive medication use.

### 2.3 Patient Selection

Patients were eligible if they had reflux symptoms at least twice weekly despite ≥ 6 months of PPI or P‐CAB therapy. Preoperative evaluation included upper endoscopy, barium study, and 24‐h multichannel intraluminal impedance‐pH (MII‐pH) monitoring after medication withdrawal. High‐resolution manometry (HRM) was performed in all patients to evaluate esophageal motility according to the Chicago Classification. Patients with major motility disorders, including achalasia and esophagogastric junction outflow obstruction, were excluded. Consequently, no patients with major motility disorders were included in the final analysis [33, 34]. Esophageal biopsies were obtained when eosinophilic esophagitis was suspected [35, 36]. GERD was diagnosed according to the Lyon Consensus 2.0 criteria, [37] incorporating acid exposure time (AET), reflux episodes, mean nocturnal baseline impedance (MNBI), and endoscopic findings. Patients with non‐pathologic reflux (AET < 4.0%, reflux episodes < 40/day, MNBI > 2500 Ω) or negative symptom correlation (symptom index < 50% or symptom association probability < 95%) were excluded. Additional exclusion criteria included prior upper gastrointestinal surgery, previous anti‐reflux interventions, major systemic illness, or contraindications to endoscopy.

**VIDEO 1:** Endoscopic hand suturing during anti‐reflux mucoplasty (ARM‐P). Video content can be viewed at https://onlinelibrary.wiley.com/doi/10.1111/den.70201.

### 2.4 Data Collection

Clinical data included demographic characteristics, GERD duration, antithrombotic use, American Society of Anesthesiologists Physical Status (ASA‐PS) classification, endoscopic findings, and MII‐pH parameters. Procedural variables and complications were recorded. Endoscopic findings were independently reviewed by two board‐certified endoscopists of the Japanese Society of Gastrointestinal Endoscopy, and any discrepancies were resolved by adjudication with a third reviewer to reach a consensus diagnosis.

### 2.5 Endoscopic Anti‐Reflux Procedure

A therapeutic gastroscope (GIF‐Q260J, GIF‐290T, or H290T; Olympus, Tokyo, Japan) with a transparent distal hood was used, with continuous CO_2_ insufflation to maintain stable visualization of the gastric cardia in retroflexion. A handmade snare‐assisted angulation booster was used to facilitate tip angulation [38] (Video 1).

### 2.6 Conventional Techniques (ARMA and ARMS)

Conventional techniques (ARMA and ARMS) involved mucosal ablation or resection of approximately three‐quarters to four‐fifths (3/4–4/5) of the gastric cardia circumference, as previously described. ARMA was performed using either a Triangle Tip Knife J (Olympus) in spray coagulation mode (50 W, Effect 2) or argon plasma coagulation (70–100 W, 1.0 L/min) with an APC probe (ERBE Elektromedizin). ARMS involved mucosal resection using either cap‐assisted endoscopic mucosal resection (EMR‐C) [39] with a large oblique cap (MAJ‐296; Olympus) and a thin snare (SD‐221 L‐25; Olympus) or endoscopic submucosal dissection (ESD).

### 2.7 Closure‐Focused Techniques (ARM‐P and ARM‐P/V)

**FIGURE 1:**
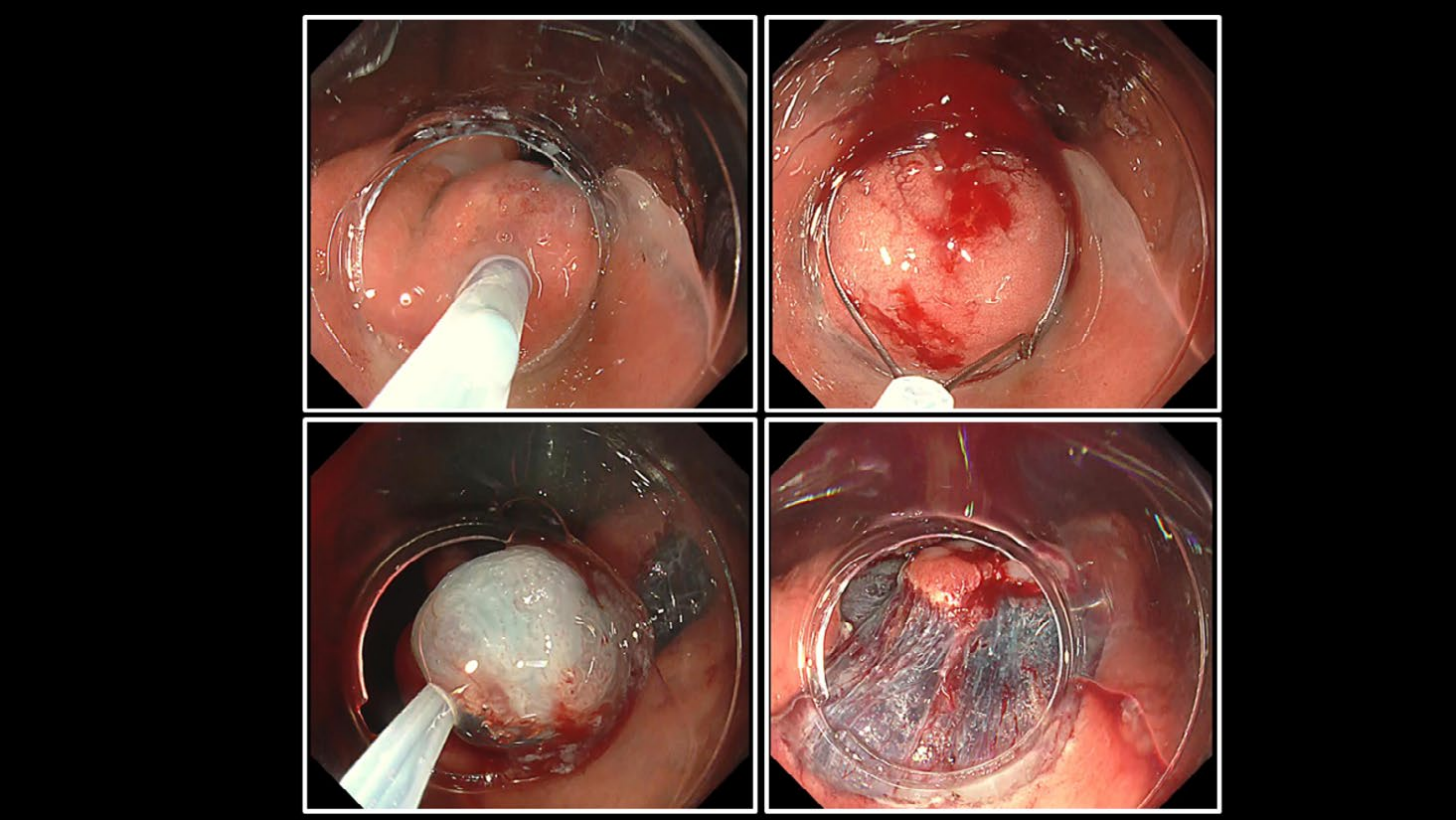
Anti‐reflux mucoplasty (ARM‐P). A submucosal injection of saline mixed with indigo carmine is administered using a 25‐gauge needle, followed by endoscopic mucosal resection with a cap (EMR‐C) along the lesser curvature. Three to four resections typically remove approximately one‐third of the cardia. The resulting mucosal defect is shown prior to closure.

**FIGURE 2:**
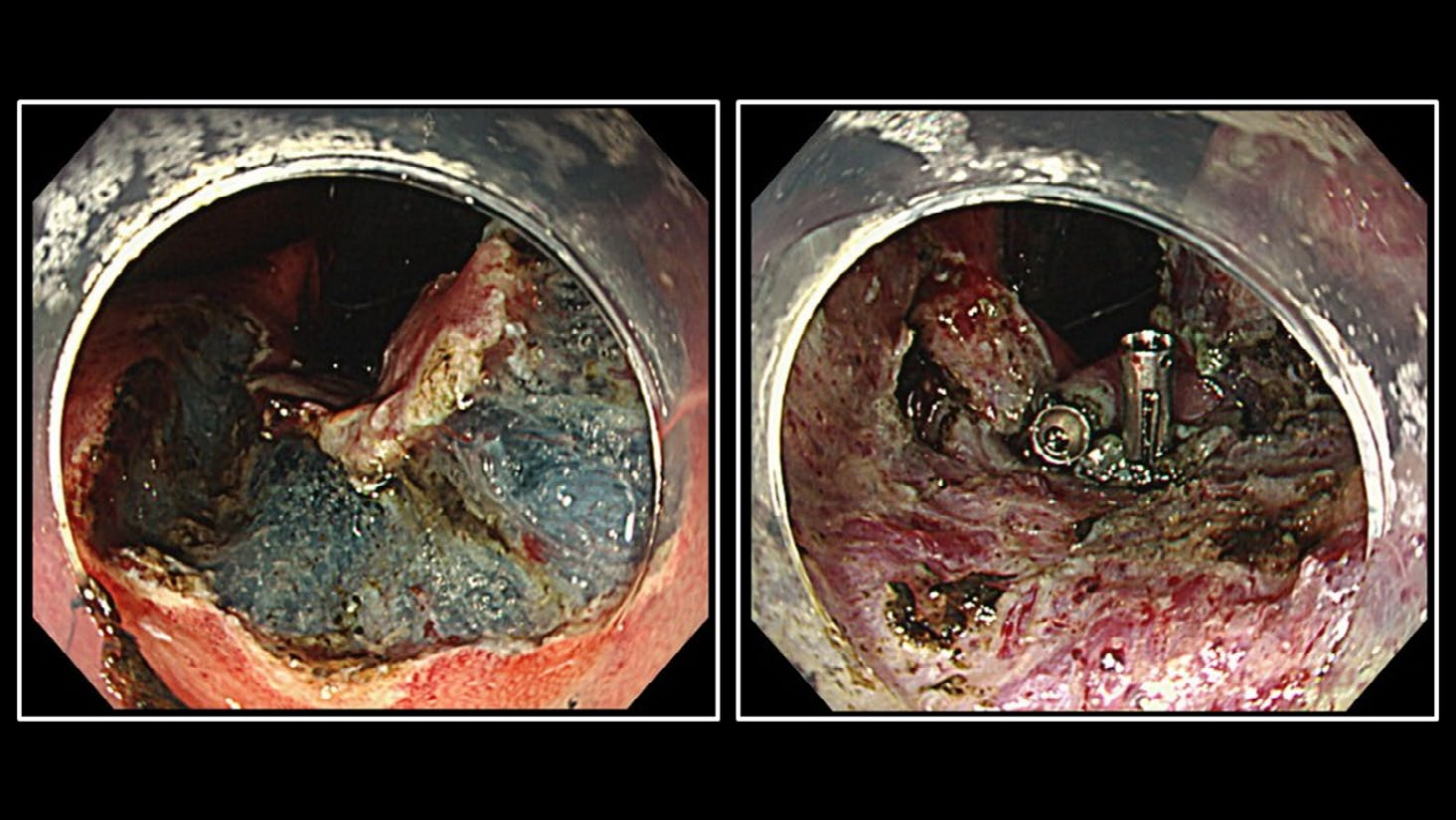
Anti‐reflux mucoplasty with valve (ARM‐P/V). Mucosal incisions are created along one‐third of the lesser curvature, and submucosal dissection is extended to form a double‐layered mucosal flap. Two to three reopenable clips are then used to anchor the flap to the muscularis propria, preserving elevation and creating a valve.

ARM‐P and ARM‐P/V were designed to reduce resection extent and enable immediate defect closure. In ARM‐P, approximately one‐third of the lesser‐curvature side of the gastric cardia was resected using EMR‐C (Figure 1). In ARM‐P/V, the same arc was resected using ESD with a Triangle Tip Knife J and an electrosurgical generator (VIO3; ERBE, Endocut I mode 1‐3‐3). The mucosal flap was anchored to the muscularis propria with reopenable clips (SureClip; Microtech) to create a reinforced mucosal valve (Figure 2). Immediate closure was performed in both techniques using loop‐clip devices (Loop‐9), [40] c clip‐with‐line systems (Loop‐10/11), [25, 41] endoloop‐assisted closure, anchor‐pronged clips (MANTIS; Boston Scientific), [42] or endoscopic hand suturing with a disposable needle holder (FG‐260Q; Olympus) and 3–0 V‐Loc sutures (Medtronic) [43]. Successful closure was defined as complete approximation of the mucosal defect without residual gaps.

### 2.8 Postoperative Management and Follow‐Up

**FIGURE 3:**
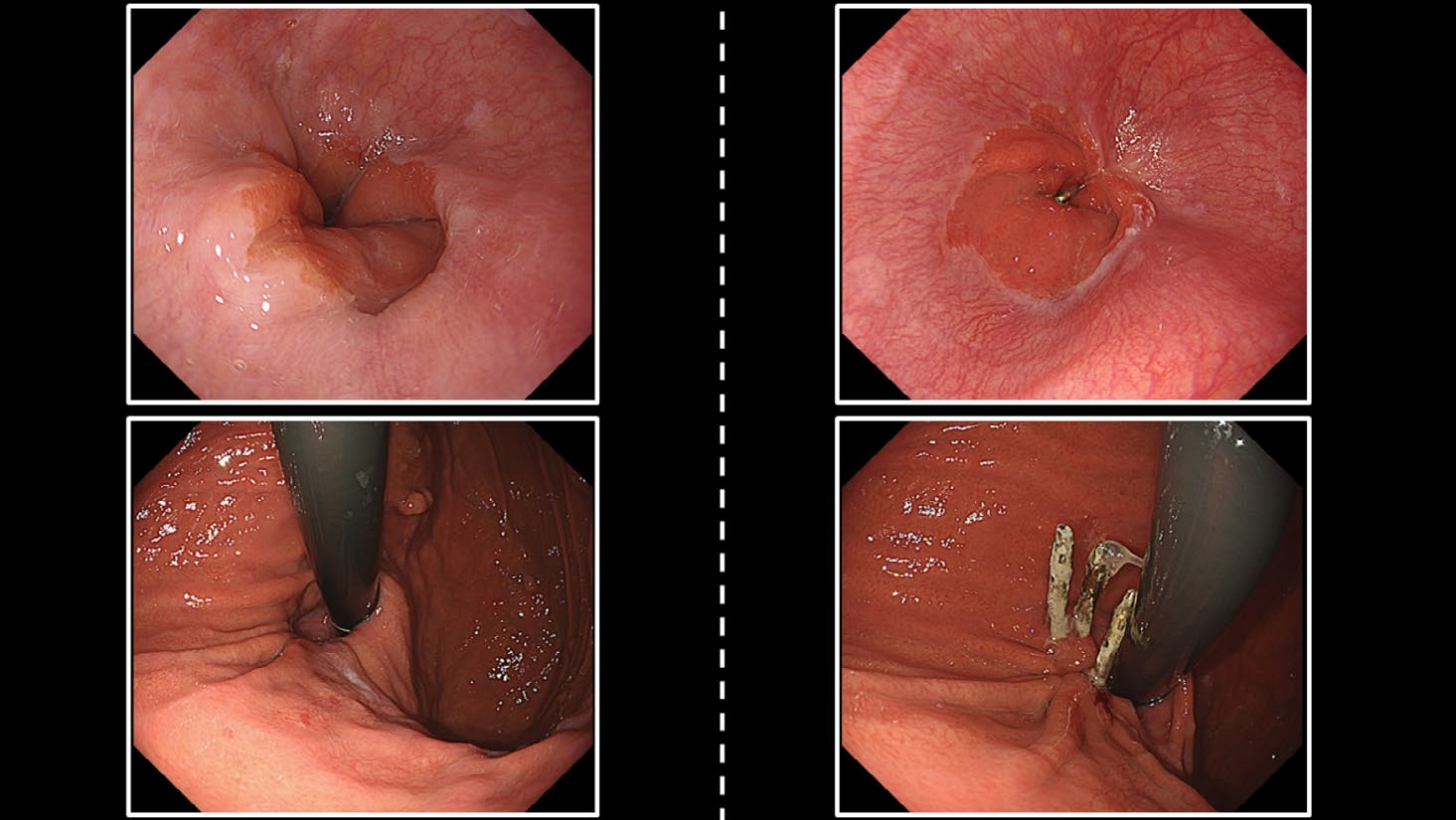
Endoscopic view before and after anti‐reflux mucoplasty (ARM‐P). Pre‐procedure endoscopy demonstrates the gastroesophageal junction prior to treatment. Follow‐up endoscopy shows a well‐developed and enhanced gastroesophageal valve following ARM‐P.

**FIGURE 4:**
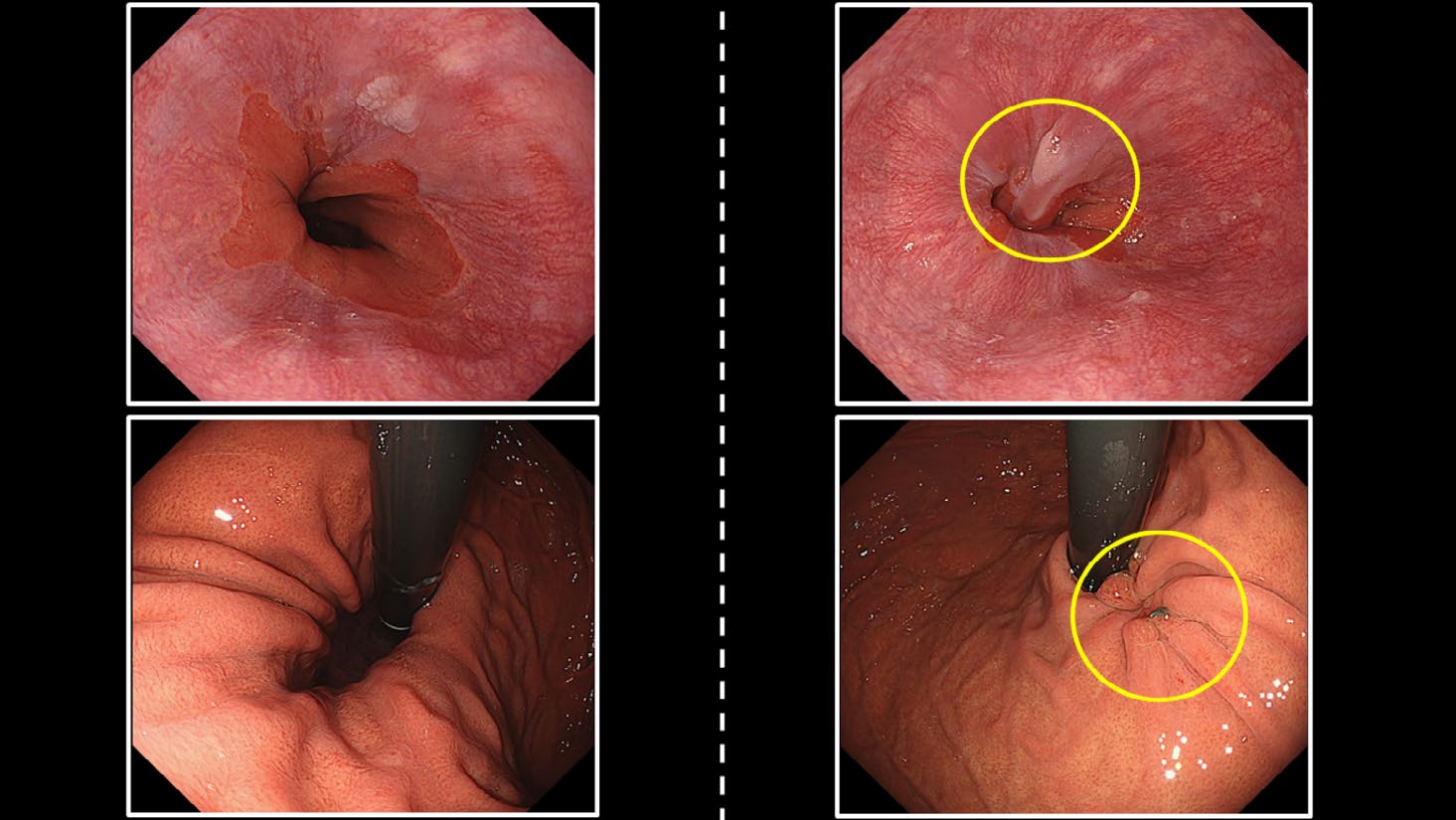
Endoscopic view before and after anti‐reflux mucoplasty with valve (ARM‐P/V). Pre‐procedure endoscopy demonstrates the gastroesophageal junction before intervention. Follow‐up endoscopy reveals a reconstructed, valve‐like structure with remodeling of the gastroesophageal flap valve. The yellow circle highlights the newly formed valve.

Postoperative endoscopy was performed on postoperative day (POD) 1 and again on POD 4 or 5. During hospitalization, patients were monitored with vital signs every 6 h and routine laboratory testing on POD 1. A clear liquid diet was initiated once no clinical or endoscopic concerns were identified, and patients were discharged after tolerating oral intake. At discharge, all patients received a PPI or P‐CAB for approximately 1 month to promote ulcer healing. The first follow‐up visit was scheduled at approximately 2 months, when upper endoscopy and validated symptom questionnaires were performed to assess mucosal healing, anatomical changes, adverse events, and reflux symptoms. These assessments were conducted by non‐blinded physicians as part of routine clinical practice. Follow‐up endoscopy (GIF‐XZ1200; Olympus) typically showed improved GEFV morphology and reduced hiatal hernia when present (Figures 3 and 4). PPI/P‐CAB discontinuation was defined as complete cessation at 2 months, and dose reduction as continuation at a lower dose than baseline. Decisions regarding therapy were made by patients based on symptom status after the washout period. Patients were followed annually with upper endoscopy and outpatient visits, and those with symptoms suggestive of stenosis were instructed to return for prompt evaluation. The primary outcome was assessed from the procedure until the last follow‐up or initiation of additional anti‐reflux intervention.

### 2.9 Statistical Analysis

Propensity score matching was used to minimize baseline differences between patients treated with conventional and closure‐focused techniques. Propensity scores were estimated using logistic regression, including age, sex, BMI, duration of GERD symptoms, antithrombotic use, ASA‐PS classification, pre‐treatment Los Angeles (LA) grade (LA‐M and LA‐N coded as 0), AET, number of reflux events, and pre‐treatment Hill classification. Patients were matched 1:1 using nearest‐neighbor matching without replacement with a caliper of 0.2 standard deviations of the logit of the propensity score. Categorical variables were expressed as frequencies and percentages, and continuous variables as mean ± standard deviation. Groups were compared using the independent *t*‐test or the chi‐squared or Fisher's exact test, as appropriate. Balance after matching was assessed using standardized differences, with values < 0.1 indicating good balance. The average treatment effect for the treated (ATET) was estimated, with closure‐focused techniques defined as the treated cohort, to evaluate the effect of ARM‐P and ARM‐P/V after adjustment for measured confounders. All analyses were performed using Stata version 14 (StataCorp), with *p* < 0.05 considered statistically significant. As this was a retrospective observational study, no a priori sample size calculation was performed, and all consecutive eligible patients during the study period were included in the analysis.

## 3 Results

### 3.1 Patient Characteristics

A total of 251 patients were evaluated before matching, including 147 treated with conventional techniques (ARMA or ARMS) and 104 treated with closure‐focused techniques (ARM‐P or ARM‐P/V). In this cohort, 110 patients had an AET > 4%, including 73 in the conventional group and 37 in the closure‐focused group. After 1:1 propensity score matching, 94 matched pairs (188 patients) were generated. Baseline characteristics were well balanced between groups, with standardized differences close to zero for all variables, including age, sex, BMI, duration of GERD symptoms, antithrombotic use, ASA‐PS classification, pre‐treatment LA grade, AET, number of reflux events, and pre‐treatment Hill classification, as shown in Table 1. In the matched cohort, procedure time was significantly longer for closure‐focused techniques than for conventional techniques (74.65 ± 25.89 vs. 49.33 ± 23.58 min; *p* < 0.001) (Table 2). Follow‐up duration remained significantly shorter in the closure‐focused group (248.63 ± 181.49 vs. 1513 ± 1144.59 days; *p* < 0.001), reflecting the more recent introduction of these techniques.

**TABLE 1:** Comparison of baseline characteristics between conventional and closure‐focused techniques (before and after matching).

	All patients				Propensity‐matched patients			
	Conventional techniques (*n* = 147)	Closure‐focused techniques (*n* = 104)	*p*	Standardized difference	Conventional techniques (*n* = 94)	Closure‐focused techniques (*n* = 94)	*p*	Standardized difference
Sex, *n* (%)^a^								
Male	84 (57.14)	64 (61.54)	0.486	0.089	37 (39.36)	37 (39.36)	1.000	0.000
Female	63 (42.86)	40 (38.46)			57 (60.64)	57 (60.64)		
Age, mean ± SD, years^a^	54.68 ± 16.16	55.41 ± 15.73	0.724	0.045	55.72 ± 15.75	54.85 ± 15.45	0.612	0.056
BMI, mean ± SD, mg/dL^a^	22.02 ± 3.94	22.28 ± 3.21	0.590	0.070	22.03 ± 3.99	22.17 ± 3.05	0.455	0.038
ASA score, *n* (%)^a^								
ASA I	74 (50.34)	40 (38.46)	0.280	0.170	44 (46.81)	37 (39.36)	0.507	0.298
ASA II	63 (42.86)	64 (61.54)			46 (48.94)	57 (60.64)		
ASA III	6 (4.08)	0 (0)			4 (4.26)	0 (0)		
ASA IV	0 (0)	0 (0)			0 (0)	0 (0)		
ASA V	0 (0)	0 (0)			0 (0)	0 (0)		
Anticoagulation use, *n* (%)^a^								
Yes	6 (4.08)	6 (5.77)	0.578	0.078	6 (6.38)	5 (5.32)	1.000	0.045
No	141 (95.92)	98 (94.23)			88 (93.62)	89 (94.68)		
Duration of GERD symptoms, mean ± SD, years^a^	5.13 ± 5.75	6.99 ± 6.73	0.021	0.298	5.83 ± 6.39	7.21 ± 6.91	0.053	0.208
LA grade classification, *n* (%)^a^								
Grade M/N	99 (67.35)	80 (76.92)	0.489	0.162	71 (75.54)	75 (79.79)	0.233	0.080
Grade A	24 (16.33)	13 (12.50)			13 (13.83)	11 (11.70)		
Grade B	17 (11.56)	8 (7.69)			7 (7.45)	6 (6.38)		
Grace C	3 (2.04)	2 (1.92)			1 (1.06)	2 (2.13)		
Grade D	4 (2.72)	1 (0.96)			2 (2.13)	0 (0)		
Hill classification, *n* (%)^a^								
Grade I	13 (8.84)	6 (5.77)	0.868	0.315	9 (9.57)	4 (4.26)	0.170	0.302
Grade II	69 (46.94)	33 (31.73)			41 (43.62)	32 (34.04)		
Grade III	57 (38.78)	57 (54.81)			39 (41.49)	50 (53.19)		
Grade IV	8 (5.44)	8 (7.69)			5 (5.32)	8 (8.51)		
GERDQ score, mean ± SD	9.29 ± 2.82	9.29 ± 2.81	0.744	0.043	9.19 ± 2.87	9.44 ± 2.74	0.378	0.087
GERD‐HRQL score, mean ± SD	31.17 ± 14.07	21.73 ± 10.26	< 0.001	0.751	32.04 ± 14.56	22.13 ± 10.37	< 0.001	0.783
FSSG score, mean ± SD	25.32 ± 9.61	23.85 ± 10.01	0.460	0.097	24.87 ± 9.55	24.71 ± 10.34	0.892	0.018
24‐h pH monitoring								
AET, mean ± SD, %	14.70 ± 23.60	5.55 ± 11.89	0.001	0.490	6.98 ± 10.55	4.80 ± 10.29	0.099	0.209
All reflux events, mean ± SD	76.00 ± 41.96	59.26 ± 27.94	0.001	0.469	65.65 ± 32.68	5.89 ± 10.45	0.147	0.233
Number of acid reflux events, mean ± SD	40.69 ± 29.71	34.44 ± 21.13	0.090	0.242	30.88 ± 23.31	33.60 ± 19.62	0.193	0.126
Number of non‐reflux acid reflux events, mean ± SD	36.40 ± 32.74	25.10 ± 18.82	0.004	0.423	34.00 ± 25.56	25.29 ± 18.83	0.021	0.388
Symptom index, mean ± SD	0.61 ± 0.39	0.51 ± 0.40	0.044	0.259	0.63 ± 0.37	0.54 ± 0.38	0.100	0.237
Symptom association probability, mean ± SD	0.65 ± 0.46	0.70 ± 0.44	0.416	0.105	0.70 ± 0.44	0.75 ± 0.41	0.781	0.106

Abbreviations: AET, acid exposure time; ASA‐PS, American Society of Anesthesiologists Physical Status; BMI, body mass index; FSSG, Frequency Scale for the Symptoms of GERD; GERD, gastroesophageal reflux disease; GERD‐HRQL, GERD‐Health Related Quality of Life Questionnaire; GerdQ, Gastroesophageal Reflux Disease Questionnaire; LA, Los Angeles; SD, standard deviation.

^a^ Variables included in the propensity score model: age, sex, BMI, duration of symptoms, anticoagulant use, ASA‐PS score, pre‐treatment LA classification, AET, number of all reflux events, and pre‐treatment Hill classification.

**TABLE 2:** Comparison of clinical outcomes between conventional and closure‐focused techniques before and after propensity score matching.

	All patients				Propensity‐matched patients			
	Conventional techniques (*n* = 147)	Closure‐focused techniques (*n* = 104)	*p*	Standardized difference	Conventional techniques (*n* = 94)	Closure‐focused techniques (*n* = 94)	*p*	Standardized difference
Procedure time, mean ± SD, min	49.30 ± 25.46	74.09 ± 26.08	< 0.001	0.962	49.33 ± 23.58	74.65 ± 25.89	< 0.001	1.022
Length of hospital stay, mean ± SD, days	5.24 ± 4.32	4.54 ± 1.15	0.108	0.222	5.40 ± 5.20	4.55 ± 1.19	0.010	0.226
Adverse events, *n* (%)								
Total^a^	43 (29.25)	6 (5.77)	< 0.001	0.647	24 (25.53)	5 (5.32)	< 0.001	0.799
Delayed bleeding	5 (3.40)	1 (0.96)	0.214	0.167	4 (4.26)	1 (1.06)	0.368	0.199
Stenosis	39 (26.53)	1 (0.96)	< 0.001	0.607	19 (20.21)	1 (1.06)	< 0.001	1.99
Others^b^	3 (2.04)	4 (3.85)	0.394	0.611	1 (1.06)	3 (3.19)	0.310	0.148
Date of first adverse events, mean ± SD	30 ± 15.58	66 ± 161.59	0.109	0.314	29.95 ± 17.65	56.71 ± 149.61	0.151	0.251
Number of balloon dilation, mean ± SD	2.48 ± 1.97	1 ± 0	< 0.001	0.611	0.90 ± 1.78	0.01 ± 0.10	< 0.001	0.707
PPI/P‐CAB post‐treatment, *n* (%)								
Discontinuation	58 (39.46)	51 (49.04)	0.355	0.151	33 (35.11)	43 (45.74)	0.266	0.366
Dose reduction	30 (20.41)	20 (19.23)			22 (23.40)	20 (21.28)		
Continuation	59 (40.14)	33 (31.73)			39 (41.49)	31 (32.98)		
Hill classification post‐treatment, *n* (%)								
Grade I	109 (74.15)	76 (73.08)	0.746	0.187	75 (79.79)	68 (72.34)	0.419	0.175
Grade II	31 (21.09)	26 (25.00)			17 (18.09)	24 (25.53)		
Grade III	67 (45.58)	2 (1.92)			2 (2.13)	2 (2.13)		
Grade IV	1 (0.68)	0 (0)			0 (0)	0 (0)		
GerdQ score post‐treatment, mean ± SD	6.75 ± 2.85	6.78 ± 2.95	0.832	0.028	6.89 ± 3.07	6.81 ± 3.02	0.506	0.253
GERD‐HRQL score post‐treatment, mean ± SD	12.56 ± 11.69	11.17 ± 9.50	0.308	0.143	12.74 ± 11.73	11.41 ± 9.79	0.712	0.123
FSSG score post‐treatment, mean ± SD	12.43 ± 7.84	13.22 ± 9.54	0.557	0.078	12.61 ± 8.25	13.64 ± 9.70	0.698	0.114
Follow‐up duration, mean ± SD, days	1599.97 ± 1144.09	246.37 ± 180.27	< 0.001	1.653	1513 ± 1144.59	248.63 ± 181.49	< 0.001	1.544

Abbreviations: FSSG, Frequency Scale for the Symptoms of GERD; GERD‐HRQL, GERD‐Health Related Quality of Life Questionnaire; GerdQ, Gastroesophageal Reflux Disease Questionnaire; P‐CAB, potassium‐competitive acid blocker; PPI, proton pump inhibitor; SD, standard deviation.

^a^ Total adverse events represent the number of patients who experienced at least one adverse event. In the conventional techniques before matching group, four patients experienced overlapping adverse events; therefore, the sum of individual event categories exceeds the total number of patients.

^b^ Other adverse events were classified according to the adverse events in GI endoscopy (AGREE) classification and corresponded to Grade II, as they required antibiotic therapy.

### 3.2 Clinical Outcomes

Adverse events differed significantly between groups. In the matched cohort, overall adverse events were significantly lower with closure‐focused techniques than with conventional techniques (5.32% vs. 25.53%; *p* < 0.001). Transient stenosis was the most frequent complication, occurring in 20.21% of patients in the conventional group but only 1.06% in the closure‐focused group (*p* < 0.001). Delayed bleeding was uncommon and did not differ significantly between groups (4.26% vs. 1.06%; *p* = 0.368). The timing of the first adverse event was similar between groups.

Symptom scores improved significantly from baseline in both groups. Post‐treatment GerdQ, GERD‐HRQL, and FSSG scores were comparable between groups after matching. Endoscopic evaluation also demonstrated marked improvement in GEFV morphology following both interventions. The proportion of patients with Hill grade I increased markedly from baseline in both groups, with approximately three‐quarters of patients achieving Hill grade I after treatment and no significant intergroup difference (*p* = 0.419). At the 2‐month evaluation, acid‐suppressive therapy was discontinued in 45.74% of the closure‐focused group and 35.11% of the conventional group, with no significant difference between groups (*p* = 0.266).

### 3.3 Average Treatment Effect on the Treated

ATET analysis demonstrated that closure‐focused techniques significantly reduced adverse outcomes (Table 3). The risk of any adverse event was 40.4% lower in the closure‐focused group (ATET −0.404; 95% CI −0.707 to −0.102; *p* = 0.009). Similarly, the risk of stenosis was 30.8% lower (ATET −0.308; 95% CI −0.564 to −0.053; *p* = 0.018), whereas the risk of bleeding did not differ significantly between groups (ATET 0.011; 95% CI −0.013 to 0.035; *p* = 0.384). Closure‐focused techniques showed a numerically higher likelihood of acid‐suppressive medication discontinuation; however, the difference was not statistically significant (ATET 0.149; 95% CI −0.010 to 0.308; *p* = 0.067). No significant differences were observed between groups in post‐treatment Hill classification or symptom scores (GerdQ, GERD‐HRQL, and FSSG).

**TABLE 3:** Average treatment effect on the treated (ATET) for closure‐focused techniques.

	Coefficient	95% Confidence interval	*p*
Adverse events			
Total	−0.404	−0.707 to −0.102	0.009
Bleeding	0.011	−0.013 to 0.035	0.384
Stenosis	−0.308	−0.564 to −0.053	0.018
Others	0.032	−0.004 to 0.068	0.080
PPI/P‐CAB discontinuation	0.149	−0.010 to 0.308	0.067
Hill classification, post‐treatment	0.096	−0.016 to 0.208	0.094
GerdQ score, post‐treatment	0.802	−0.095 to 1.700	0.080
GERD‐HRQL score, post‐treatment	1.957	−0.844 to 4.757	0.171
FSSG score, post‐treatment	0.615	−2.890 to 4.121	0.731

Abbreviations: FSSG, Frequency Scale for the Symptoms of GERD; GERD‐HRQL, GERD‐Health Related Quality of Life Questionnaire; GerdQ, Gastroesophageal Reflux Disease Questionnaire; P‐CAB, potassium‐competitive acid blocker; PPI, proton pump inhibitor; SD, standard deviation.

## 4 Discussion

This study demonstrated improved safety while preserving therapeutic effectiveness through a propensity score–matched comparison of conventional and closure‐focused endoscopic anti‐reflux techniques. After adjustment, closure‐focused therapy showed a markedly improved safety profile while maintaining comparable short‐term symptom control, anatomical restoration, and rates of acid‐suppressive medication discontinuation.

The primary safety finding was a substantially lower incidence of adverse events with closure‐focused techniques. In the matched cohort, overall adverse events occurred in 5.32% of the closure‐focused group versus 25.53% of the conventional group. Treatment effect analyses confirmed these findings, demonstrating a 40.4% reduction in overall adverse events and a 30.8% reduction in stenosis. These results are consistent with previous reports showing that conventional techniques, such as ARMA and ARMS, carry a notable risk of stenosis (7%–14.4%) [14, 16, 18, 19, 20]. Because conventional approaches rely on secondary healing, fibrosis and contracture may lead to luminal narrowing. In contrast, immediate closure appears to stabilize early wound healing and limit excessive remodeling, thereby reducing clinically significant stenosis.

Delayed bleeding outcomes also supported the safety advantage of closure‐focused therapy. Although the difference was not statistically significant, the absolute incidence was lower in the closure‐focused group. Notably, no delayed bleeding occurred after discharge, whereas conventional techniques have been associated with bleeding around postoperative day 10 [16]. The single bleeding event in the closure‐focused cohort was detected on postoperative day 1, suggesting that immediate closure may reduce ulcer exposure and shorten the bleeding risk period. Although closure‐focused techniques required longer procedure times due to mucosal closure, this did not increase complication rates.

From a clinical management perspective, the low incidence of adverse events observed in this study, particularly the minimal rate of delayed bleeding, suggests that less intensive postoperative management strategies may be feasible. In selected patients, shorter hospitalization or even day‐case procedures could be considered, which may further enhance the clinical applicability and cost‐efficiency of endoscopic anti‐reflux therapy. However, because closure‐focused techniques were newly introduced during the study period, a standardized inpatient management protocol was maintained to ensure safety during early clinical implementation. Future prospective studies are warranted to evaluate the feasibility of outpatient management and to formally assess cost‐effectiveness.

Beyond safety, closure‐focused techniques demonstrated functional outcomes comparable to conventional approaches. At 2 months, discontinuation of acid‐suppressive therapy occurred in 45.74% of patients in the closure‐focused group and 35.11% in the conventional group (*p* = 0.266), with no significant difference confirmed by ATET analysis, indicating equivalent functional efficacy. Notably, recent prospective sham‐controlled studies have reported no significant benefit of ARMA compared with sham procedures, highlighting ongoing controversy in this field. However, our findings demonstrated meaningful symptomatic and anatomical improvement, suggesting that differences in clinical context may influence treatment outcomes [23]. Symptomatic and anatomical outcomes were also comparable between groups. GerdQ, GERD‐HRQL, and FSSG scores improved significantly from baseline in both groups, with no significant intergroup differences after matching. Endoscopic assessment showed marked improvement in GEFV morphology, with approximately three‐quarters of patients achieving Hill grade I in both groups. These findings indicate that both conventional and closure‐focused approaches effectively enhance the anti‐reflux barrier.

This study has several strengths, including comprehensive physiological and endoscopic baseline assessment, propensity score matching to reduce confounding, and evaluation of both symptomatic and anatomical outcomes. However, several limitations should be noted. First, this was a retrospective single‐center study, which may limit generalizability. In addition, because closure‐focused techniques were introduced later in the study period, potential calendar‐time effects and learning‐curve influences on procedural efficiency and adverse event outcomes cannot be completely excluded. Although all procedures were performed by board‐certified expert endoscopists with extensive experience in therapeutic endoscopy, which likely minimized operator‐related variability, residual bias related to temporal changes in practice remains possible. In addition, outcome assessments were performed by non‐blinded physicians, which may have introduced potential bias, particularly in the evaluation of subjective endpoints. Second, although this study demonstrated the overall benefit of closure‐focused techniques, it did not allow method‐specific comparisons, as multiple closure techniques were used within the closure‐focused group. Differences among closure methods may have influenced clinical outcomes, and the effects of individual techniques could not be separately evaluated. Third, stenosis was defined as dysphagia requiring endoscopic balloon dilation, which reflects clinically significant stenosis but may introduce some subjectivity. Fourth, postprocedural MII‐pH monitoring was not systematically performed, limiting objective assessment of reflux control. Finally, all procedures were performed by experienced endoscopists, which ensured procedural consistency but may limit generalizability. Prospective multicenter studies are needed to confirm these findings.

## 5 Conclusion

Closure‐focused techniques significantly reduce adverse events, particularly transient stenosis, without compromising symptomatic or anatomical outcomes. Comparable rates of acid‐suppressive medication discontinuation support their clinical effectiveness. These findings suggest that closure‐focused techniques represent a safer refinement of endoscopic anti‐reflux therapy; however, prospective comparative studies are needed for validation.

## Author Contributions

K.Y., H.I., Ko.S., and N.T.V.R. led and designed the study. Ko.S. and N.T.V.R. contributed to the statistical analysis. K.Y., Y.T., I.T., and Ka.S. contributed to data collection and evaluated the endoscopic findings. K.Y. was responsible for drafting, revising, and finalizing the manuscript. H.I., Ko.S, Y.T., K.U., Y.N., I.T., M.T., Ka.S., and N.T.V.R. critically reviewed the manuscript and contributed to the discussion. All authors read and approved the final manuscript.

## Funding

The authors have nothing to report.

## Ethics Statement

The study received approval by the Institutional Review Board of the Showa Medical University (approval no.: 2025‐0397).

## Consent

The authors have nothing to report.

## Conflicts of Interest

H.I. is an advisor of Olympus Corporation and Top Corporation. The other authors declare no conflicts of interest for this article.

## Data Availability

The data that support the findings of this study are available from the corresponding author upon reasonable request.
